# Supplementary material for: Causal Relationship between Aging and Anorexia Nervosa: A White-Matter-Microstructure-Mediated Mendelian Randomization Analysis
Source: Biomedicines. 2024 Aug 16;12(8):1874. doi: 10.3390/biomedicines12081874 (PMC11351342; doi:10.3390/biomedicines12081874)
Supplement: Supplementary file 1 [file biomedicines-12-01874-s001.zip › Supplementary Figure/Figure S5/Scatter plot of MR analysis for ubm-b-1946 to AN.pdf]

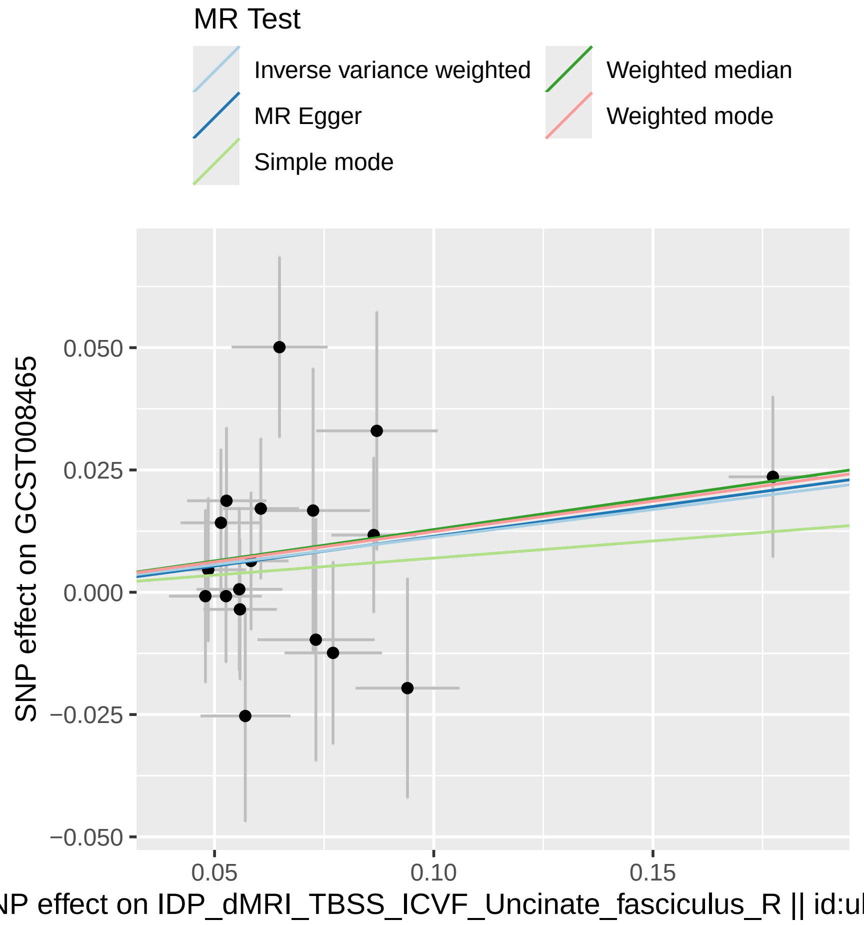

MR Test
Inverse variance weighted
MR Egger
Simple mode
Weighted median
Weighted mode
0.050
0.025
0.000
−0.025
−0.050
0.05
0.10
0.15
SNP effect on GCST008465
NP effect on IDP_dMRI_TBSS_ICVF_Uncinate_fasciculus_R || id:ul
